# Sodium Ferulate Attenuates Lidocaine-Induced Corneal Endothelial Impairment

**DOI:** 10.1155/2018/4967318

**Published:** 2018-07-08

**Authors:** Guojian Jiang, Tingjun Fan

**Affiliations:** Laboratory for Corneal Tissue Engineering, College of Marine Life Sciences, Ocean University of China, Yushan Road No. 5, Qingdao 266003, China

## Abstract

The introduction of intracameral anaesthesia by injection of lidocaine has become popular in cataract surgery for its inherent potency, rapid onset, tissue penetration, and efficiency. However, intracameral lidocaine causes corneal thickening, opacification, and corneal endothelial cell loss. Herein, we investigated the effects of lidocaine combined with sodium ferulate, an antioxidant with antiapoptotic and anti-inflammatory properties, on lidocaine-induced damage of corneal endothelia with *in vitro* experiment of morphological changes and cell viability of cultured human corneal endothelial cells and *in vivo* investigation of corneal endothelial cell density and central corneal thickness of cat eyes. Our finding indicates that sodium ferulate from 25 to 200 mg/L significantly reduced 2 g/L lidocaine-induced toxicity to human corneal endothelial cells, and 50 mg/L sodium ferulate recovered the damaged human corneal endothelial cells to normal growth status. Furthermore, 100 mg/L sodium ferulate significantly inhibited lidocaine-induced corneal endothelial cell loss and corneal thickening in cat eyes. In conclusion, sodium ferulate protects human corneal endothelial cells from lidocaine-induced cytotoxicity and attenuates corneal endothelial cell loss and central corneal thickening of cat eyes after intracameral injection with lidocaine. It is likely that the antioxidant effect of sodium ferulate reduces the cytotoxic and inflammatory corneal reaction during intracameral anaesthesia.

## 1. Introduction

In cataract surgery, traditional retrobulbar and peribulbar anaesthesia is associated with dangerous complications such as globe perforation, retrobulbar hemorrhage, optic nerve trauma, brainstem anaesthesia, and extraocular muscle injury [1, 2]. Topical anaesthesia has limitations, such as inadequate motor and sensory anaesthesia, insufficient analgesia, and increased intraoperative pain [3–5]. Intracameral anaesthesia acts directly on the iris and ciliary body, providing a significant decrease in pain and discomfort during intraocular procedures of cataract surgery [4]. Therefore, intracameral and topical anaesthesia when used in combination avoids the risks associated with retrobulbar and peribulbar blocks and provides another option for analgesia in ocular surgery [3–7].

Lidocaine (LD) has become a commonly used anaesthetic agent because of its inherent potency, rapid onset, tissue penetration, and efficiency [4]. However, intracameral LD causes adverse events such as corneal thickening, opacification, and significant corneal endothelial cell loss [8, 9]. In addition, increasing evidence shows that LD exerts cytotoxicity to corneal endothelial cells in a time- and dosage-dependent manner [9–13]. For example, higher concentrations cause necrosis by disruption of membranes, and lower concentrations induce apoptosis mainly by excessive reactive oxygen species (ROS) production via the mitochondrial pathway [10–17]. Methods to reduce the cytotoxicity, especially to relieve oxidative stress of LD, are important to protect the corneal endothelium and guarantee clinically safe administration during cataract surgery.

Sodium ferulate (SF), a sodium salt of ferulic acid (3-methoxy-4-hydroxy-cinnamate sodium), acts as a potent antioxidant by scavenging ROS and enhancing the cell stress response through the upregulation of cytoprotective systems, such as heme oxygenase-1, superoxide dismutase, catalase, and Hsp70 [18]. Furthermore, SF inhibits the expression and/or activity of enzymes, including inducible nitric oxide synthase, caspases, and cyclooxygenase-2, as well as the activation of JNK [18–23].

In the present study, both *in vitro* and *in vivo* experiments were designed to investigate the cytoprotective effects of SF on LD-induced corneal endothelial dysfunction and to determine if the clinical intracameral SF administration protects the corneal endothelia during anaesthesia.

## 2. Materials and Methods

### 2.1. Reagents

LD, dimethyl sulfoxide (DMSO), and 3-[4,5-dimethylthiazol-2-yl]-2,5-dipheny-l tetrazolium bromide (MTT) were purchased from Sigma-Aldrich (St. Louis, MO, USA). SF was the product of Qufu Hongly Chemical Industry Co. Ltd. (Shandong, China). Fetal bovine serum (FBS) was from Hyclone (Logan, Utah, USA). Dulbecco's modified Eagle media : Ham's nutrient mixture F-12 (1 : 1) medium (DMEM/F12) was obtained from Invitrogen (Carlsbad, CA, USA).

### 2.2. Cell Culture

Human corneal endothelial (HCE) cells at 120 passages established in our laboratory were maintained and cultured in DMEM/F12 media containing 10% FBS (10% FBS-DMEM/F12) at 37°C and 5% CO_2_ [24].

### 2.3. Morphological Observation of HCE Cells

HCE cells were seeded onto a 24-well culture plate at a density of 5 × 10^4^ cells per well and cultured in 10% FBS-DMEM/F12 at 37°C and 5% CO_2_. To evaluate the effects of SF on LD-induced cytotoxicity, the HCE cells at a logarithmic phase were divided into seven groups and treated with LD and SF dissolved in 10% FBS-DMEM/F12 medium during which cell morphology was monitored for 24 h (Table 1). Subsequently, the growth medium was replaced with 10% FBS-DMEM/F12 containing 50 mg/L SF, and the HCE cells were observed 48 h later to determine if damaged cells can be recovered (Table 1). HCE cells at a logarithmic phase were also divided into three groups and treated with different combinations of LD and SF in 10% FBS-DMEM/F12 medium for 4 h (Table 2). Then, the growth medium was removed entirely, and 10% FBS-DMEM/F12 was supplemented (Table 2). The HCE cells were observed after 24 h to determine if 10% FBS-DMEM/F12 medium without SF allows the recovery of the damaged cells. Cell morphology and growth were monitored under an Eclipse TS100 inverted light microscope (Nikon, Tokyo, Japan).

### 2.4. Determination of Cell Viability by the MTT Assay

HCE cell viability was measured after treatment with LD and SF by using the MTT assay. Cells were seeded onto a 96-well cell plate at a density of 1 × 10^4^ cells per well and cultured 48 h prior to treatments as described above. The growth medium was then removed, and 100 *μ*L fresh medium containing 1.1 mM MTT was added prior to incubation for 4 h at 37°C in the dark. The medium was discarded, and 100 *μ*L DMSO was added. Cell viability was determined by measuring the optical density values of samples using a microplate reader (Multiskan GO; Thermo Scientific, Waltham, MA, USA) at an absorption wavelength of 590 nm.

### 2.5. In Vivo Experimental Procedure

All of the animals in the present study were 5-year-old male domestic cats (*Felis catus*) weighing 3.6 ± 0.5 kg. Fifteen cats were acclimatized to the laboratory environment for 7 days; then, the health of their corneal endothelia was determined by ophthalmic examination including corneal endothelial cell density (CECD), mean cell area, coefficient of variation in cell size, percentage hexagonality, and central corneal thickness (CCT). Nine cats with healthy corneal endothelia were chosen and divided randomly into 3 groups. The anterior chamber of the eye was entered using a 30-gauge needle from the supero-temporal limbal area, and 150 *μ*L of aqueous humor was aspirated and replaced with 150 *μ*L of sterile solutions containing LD in combination with SF at the concentrations shown in Table 3. The drugs were prepared in fortified balanced salt solution (BSS) and BSS without drugs as control. CECD and CCT for each group were assayed at days 0, 5, 9, and 12 after injection of drugs. Before ophthalmic examination and intracameral injection, the cats were anaesthetized using Zoletil (Virbac France, 10 mg/kg, consisting of tiletamine and zolazepam) intramuscularly. Ophthalmic examination was carried out with noncontact specular microscopy (SP-3000P: Topcon Corporation, Tokyo, Japan). The individual differences in CECD and CCT amongst the cats were determined as percentage of CECD and percentage of CCT. The percentage of CECD was calculated as “CECD(%) = (CECD of experimental group/CECD of the same eye before exposure to drugs) × 100,” and the percentage of CCT was calculated as “CCT(%) = (CCT of experimental group/CCT of the same eye before exposure to drugs) × 100.” All procedures described in this study were conducted according to the guidelines in the Association for Research in Vision and Ophthalmology (ARVO) Statement for the Use of Animals in Ophthalmic and Vision Research and were approved by the institutional Ethics Committee of Animal Care and Experimentation (approval number SD-SYKY-2014-021).

### 2.6. Statistical Analysis

The results were presented as mean ± standard deviation (SD) from three independent experiments and analyzed by one-way analysis of variance (ANOVA) followed by post hoc test of Student's *t-*test with Bonferroni's correction. *P* values less than 0.05 were considered statistically significant (^∗^*P* < 0.05, ^∗∗^*P* < 0.01).

## 3. Results

### 3.1. Drug-Induced Morphological Changes of HCE Cells

**Figure 1:**
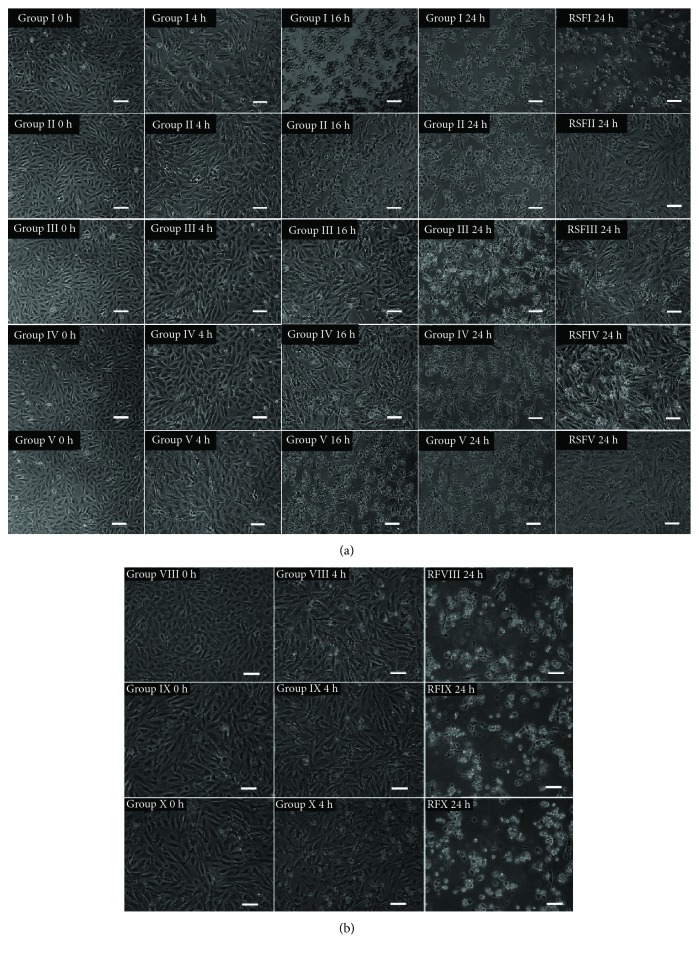
(a) Morphological changes in HCE cells after treatment with LD and SF for 24 h followed by incubation with 50 mg/L SF. The treatments and observation times are shown at the top left of each photograph. One representative photograph from three independent experiments is shown. Groups I–V: HCE cells were treated with 2 g/L LD in combination with 0, 25, 50, 100, and 200 mg/L SF, respectively. RSFI–RSFV: incubation of groups I–V for 24 h followed by treatment with 50 mg/L SF. Typical morphological features of HCE cells are shrinkage, rounding, cytoplasmic vacuolization, and detachment from substratum. Scale bar: 50 *μ*m. (b) Morphological changes in HCE cells after LD and SF treatment for 4 h followed by incubation in 10% FBS-DMEM/F12. The treatment and observation times are shown in the top left of each photograph. One representative photograph from three independent experiments is shown. Groups VIII–X: HCE cells were treated, respectively, with 2 g/L LD in combination with 0, 50, and 100 mg/L SF in 10% FBS-DMEM/F12. RSFVIII–RSFX: treatment of GVIII–GX for 4 h followed by treatment with 10% FBS-DMEM/F12. Scale bar: 50 *μ*m.

The use of LD at 2 g/L was determined previously [10]. Morphological changes evaluated were cell shrinkage, cell rounding, cytoplasmic vacuolization, and detachment from the culture matrix, which are all similar to changes that occur in apoptotic cells [10, 25–28]. The HCE cells in group I (Table 1) began shrinking at 4 h whereas group II, group III, group IV, and group V cells exhibited no obvious morphological changes (Figure 1(a)). At 16 h, HCE cells in group I exhibited clearer apoptotic characteristics than those in groups II–V. In addition, the cell density in group III and group IV was higher than that in groups I, II, and V (Figure 1(a)). At 24 h, most of the HCE cells in groups II–V became rounding and showed cytoplasmic vacuolization, whereas cells in group I which were at low density began to detach and float in the medium (Figure 1(a)). Twenty-four hours after the medium was replaced with 10% FBS-DMEM/F12 containing 50 mg/L SF, cells in groups II–V recovered to normal morphology and formed confluent monolayers. However, almost all of the cells in group I were floating in the medium (Figure 1(a)). 10% FBS-DMEM/F12 without SF did not prevent LD-induced apoptosis (Figure 1(b)). The cells in group VI, group VII, and control remained in a confluent monolayer, and their appearance was normal throughout the experiment indicating that SF concentrations of 200 mg/L and less were not toxic to HCE cells (not shown).

### 3.2. HCE Cell Viability

**Figure 2:**
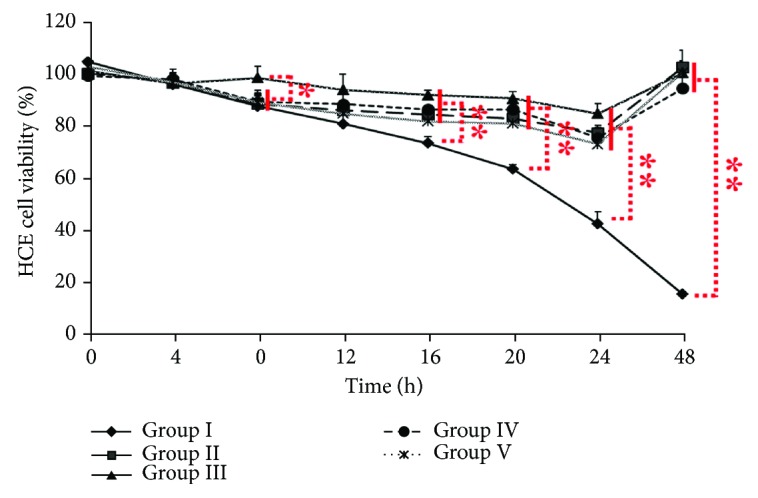
Viability of HCE cells cultured *in vitro* after treatment with LD and/or SF. ♦: group I: HCE cells were treated with 2 g/L LD alone for 24 h followed by treatment with 50 mg/L SF in 10% FBS-DMEM/F12 from 24 to 48 h; ■: group II: HCE cells were treated with 2 g/L LD in combination with 25 mg/L SF followed by treatment with 50 mg/L SF in 10% FBS-DMEM/F12 for 24 h followed by treatment with 50 mg/L SF in 10% FBS-DMEM/F12 from 24 to 48 h; ▲: group IIII: HCE cells were treated with 2 g/L LD in combination with 50 mg/L SF for 24 h followed by treatment with 50 mg/L SF in 10% FBS-DMEM/F12 from 24 to 48 h; ●: group IV: HCE cells were treated with 2 g/L LD in combination with 100 mg/L SF for 24 h followed by treatment with 50 mg/L SF in 10% FBS-DMEM/F12 from 24 to 48 h; **✳**: group V: HCE cells were treated with 2 g/L LD in combination with 200 mg/L SF for 24 h followed by treatment with 50 mg/L SF in 10% FBS-DMEM/F12 from 24 to 48 h. ^∗∗^*P* < 0.01, ^∗^*P* < 0.05.

The viability of HCE cells in groups I to V treated with LD and SF was examined by MTT. From 0 to 4 h, variations in the viability of cells in groups I–V were not significant (Figure 2) (*P* > 0.05). However, the cell viability in groups I, II, IV, and V decreased significantly at 8 h postexposure (*P* < 0.05) but not in group III (Figure 2). From 16 to 24 h, the viability of cells in groups II, III, IV, and V was significantly higher than that in group I (Figure 2) (*P* < 0.01). At 24 h, the viability of cells in group I was significantly less than the viability of cells in groups II, III, IV, and V (Figure 2) (*P* < 0.01). At 48 h, which was 24 h after growth medium was replaced with 10% FBS-DMEM/F12 containing 50 mg/L SF, the viability of cells in groups II–V increased significantly (*P* < 0.01), but the cell viability in group I decreased significantly (*P* < 0.01) (Figure 2). During the experiment, the cell viability values in group VI, group VII, and control did not change significantly (not shown).

### 3.3. Variations of CECD and CCT of the Cat Cornea after Intracameral Injection

**Figure 3:**
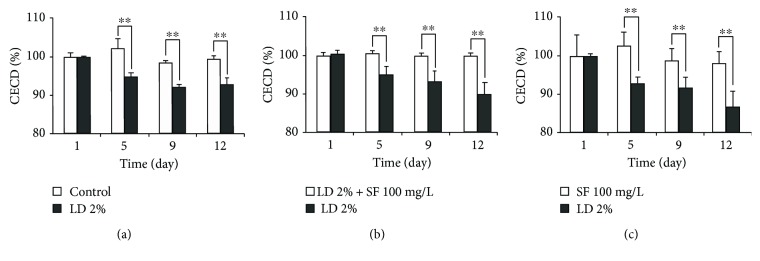
Variation in CECD of cat eyes after intracameral injection with LD and/or SF. (a) Comparison of CECD in group XI between the left eye intracamerally injected with BSS and the right eye intracamerally injected with 2% LD. (b) Comparison of CECD in group XII between the left eye intracamerally injected with 2% LD and 100 mg/mL SF and the right eye intracamerally injected with 2% LD. (c) Comparison of CECD in group XIII between the left eye intracamerally injected with 100 mg/L SF and the right eye intracamerally injected with 2% LD. ^∗∗^*P* < 0.01.

After intracameral injection of 2% LD, the percentages of CECD of the cat cornea decreased significantly from 100 ± 0.4% at day 0 to 94.2 ± 0.2% at day 12 (*P* < 0.01) (Figure 3(a)). In group XI, 2% LD treatment decreased the percentages of CECD significantly from day 0 to day 9 after which the percentages of CECD were stable from day 9 to day 12 (Figure 3(a)). The percentages of CECD of the left eye in group XII after intracameral injection of 2% LD combined with 100 mg/L SF and in group XIII after intracameral injection of 100 mg/L SF remained stable from day 0 to day 12 (*P* > 0.05), which were significantly higher than those of the right eye intracamerally injected with 2% LD at day 9 and day 12, respectively (*P* < 0.01) (Figures 3(b) and 3(c)).

**Figure 4:**
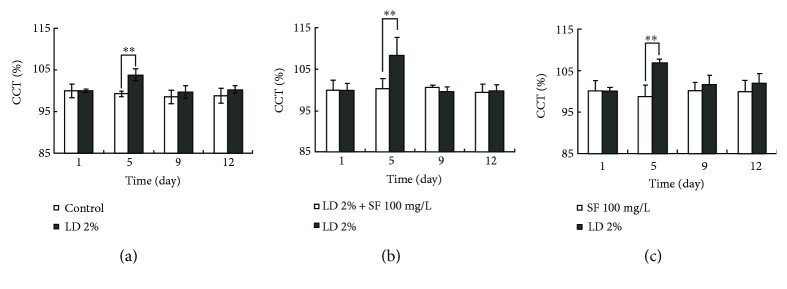
Central corneal thicknesses (CCT) of the cat eye after intracameral injection with LD and/or SF. (a) Comparison of CCT in group XI between the left eye intracamerally injected with BSS and the right eye intracamerally injected with 2% LD. (b) Comparison of CCT in group XII between the left eye intracamerally injected with 2% LD and 100 mg/mL SF and the right eye intracamerally injected with 2% LD. (c) Comparison of CCT in group XIII between the left eye intracamerally injected with 100 mg/L SF and the right eye intracamerally injected with 2% LD. ^∗∗^*P* < 0.01.

After intracameral injection of 2% LD, the percentages of CCT in the right eye of group XI increased at day 5, decreased significantly at day 9 (*P* < 0.01), and then remained stable from day 9 to day 12 (*P* > 0.05) (Figure 4(a)). The percentages of CCT of the left eye in group XII after intracameral coinjection of 2% LD combined with 100 mg/L SF and in group XIII after intracameral injection of 100 mg/L SF remained stable from day 0 to day 12 (*P* > 0.05); these values were significantly lower than those of the right eye intracamerally injected with 2% LD at day 5 (*P* < 0.01) (Figures 4(b) and 4(c)).

## 4. Discussion

LD triggers apoptosis in several cellular models by impairment of respiratory chain functions resulting in reduced ATP production, depolarization of the mitochondria, loss of mitochondrial membrane potential (*∆*Ψ), and overproduction of intracellular ROS [10, 17, 29–34]. The increased production of ROS correlates with decreased cell viability after exposure to LD, and ROS plays a vital role in inducing apoptosis of corneal cells and corneal endothelial dysfunction [34–37]. Our previous studies indicated that LD exposure to HCE cells activates caspase-3, caspase-8, and caspase-9, suggesting that the LD-induced caspase-8/9/3 pathway is influenced by the production of ROS which involves the activation of caspase-8 [10, 38, 39].

Previously, the cell morphological changes and MTT were employed to determine the apoptosis of *in vitro* cultured HCE cells, and Halilovic et al. reported the relations of the morphology of the HCE cell with its apoptotic degree [10, 25–28]. Our present study showed that 2 g/L LD initiated damage in HCE cells at 4 h postexposure which is similar to the results of previous studies [10]. Therefore, in the present study, the antioxidant SF was tested for its ability to protect corneal endothelial cells from oxidative damage caused by LD. SF has a high antioxidant potential as it donates electrons to quench free radicals and induces upregulation of many phase-2 detoxifying and antioxidant enzymes which are mediated by the NF-E2-related factor (Nrf2) signaling pathway [18, 22, 40, 41]. Furthermore, SF binds to cytochrome c to prevent assembly of the apoptosome and downregulates caspase-8 and caspase-3 [22, 42]. The present study indicates that SF delays LD-induced shrinkage of HCE cells and limits LD-induced downregulation of HCE cell viability. Upon incubation with SF after LD exposure, cells recovered and formed confluent monolayers but this did not occur with cells exposed to LD alone. Moreover, incubation in growth medium without SF did not allow recovery of HCE cells exposed to either LD or LD and SF (Figure 1(b)). The results suggest that SF reduces the LD-induced damage in HCE cells thereby allowing time for cell recovery, growth, and monolayer formation. SF at 100 mg/L significantly reduced the cytotoxic effects of LD at 2 g/L on HCE cells *in vitro*. These *in vitro* data lead to the assumption that LD has similar effects *in vivo*. LD induces excessive ROS generation in the hippocampus and amygdala of adult rats and apoptosis in rabbit corneal endothelial cells after intracameral injections [9, 12, 33]. The *in vivo* studies in cat eyes described herein demonstrated that the endothelial cell densities of the cat cornea decreased significantly after intracameral injection of 2% LD. On the basis of these findings, 100 mg/L SF in combination with 2% LD was selected as a clinically relevant dosage for intracameral injection of cat eyes. The endothelial cell densities of the cat cornea after intracameral injection of LD and SF were significantly higher than those treated with LD alone, and SF had no adverse effect on cat corneal endothelial cells. Therefore, SF is likely to reduce LD-induced cytotoxicity in corneal endothelial cells because it offsets ROS generation. Additionally, SF probably employs its ROS-scavenging systems to inhibit the activation of caspase-8-, caspase-3-, and cytochrome c-induced apoptosis by binding and enhancing the stability of cytochrome to prevent assembly of the apoptosome.

CCT evaluation is becoming increasingly important in ophthalmic practice, providing information on eyes affected by corneal ectasia [43]. There were no significant differences in corneal thickness in dogs and New Zealand white rabbits after intracameral injection of preservative-free LD at 2% [44, 45]. Moreover, a preservative-free 0.5% solution of LD has no adverse effect on the corneal thickness of human eyes [46]. However, there is increasing evidence for a relationship between intracameral injection of LD and corneal thickening. For example, anterior chamber injection of unpreserved LD causes thickening of the cornea and opacification of eyes in pigmented rabbits [47, 48].

LD-induced CCT thickening is dose-dependent and differs with species. LD-induced CCT thickening is caused by oxidative stress arising from corneal edema inflammation by excessive generation of ROS. Schellini et al. demonstrated that intracameral injection of 2% LD caused rabbit corneal edema, and Kim et al. showed that LD causes a transient endothelial cell edema in the *in vitro* perfused endothelia of human and rabbit corneas [11, 49]. In the present study, LD significantly increased CCT of the cat eye after intracameral injection and then it gradually returned to normal. SF inhibits LD-induced corneal thickening in cat eyes, which may be due to the antioxidant activity of SF. Multiple studies have shown that SF relieves inflammation caused by oxidative stress through inhibiting the activity of NF-*κ*B, the expressions of cyclooxygenase-2 and iNOS, and the contents of prostaglandin E2 and NO [22, 50]. In further study, we will firstly investigate the role of SF in the inhibition of the production of ROS, activation of caspases, and activation of NF-*κ*B in LD-impaired HCE cells to explore the mechanism of SF rescuing the oxidative-damaged HCE by antiapoptosis and anti-inflammation.

## 5. Conclusion

This study demonstrated that SF prevents LD-induced cytotoxicity in *in vitro* cultured HCE cells and attenuates the loss of corneal endothelial cell and CCT thickening of the cat eye after intracameral injection with LD. Antioxidant therapy using SF may be effective in reducing the cytotoxic and inflammatory corneal reaction during topical and intracameral anaesthesia with LD.

## Figures and Tables

**Table 1:** HCE cells were treated with LD in combination with SF and recovered by SF (*n* = 3).

Groups	Control	I	II	III	IV	V	VI	VII	RSF I–V
LD con. (g/L)	0	2					0		
SF con. (mg/L)	0	0	25	50	100	200	200	50	50
Time of observation (h)	0, 4, 8, 12, 16, 20, 24								48
Medium	10% FBS-DEME/F12								

Note: the HCE cells in groups I–V were treated with 2 g/L LD in combination with different concentrations of SF for 24 hours, respectively, and subsequently were recovered by 50 mg/L SF correspondently for 24 hours. RSF I–V: the HCE cells in groups I–V were recovered by SF 50 mg/L.

**Table 2:** HCE cells were treated with LD in combination with SF and recovered by medium (*n* = 3).

Groups	Control	VIII	IX	X	RSF VIII–X
LD con. (g/L)	0	2			0
SF con. (mg/L)	0	0	50	100	0
Time of observation (h)	0, 4				24
Medium	10% FBS-DEME/F12				

Note: the HCE cells in groups VIII–X were treated with 2 g/L LD in combination with different concentrations of SF for 4 hours, respectively, and subsequently were recovered by 10% FBS-DEME/F12 correspondently for 24 hours. RSF VIII–X: the HCE cells in groups VIII–X were cultured in 10% FBS-DEME/F12 without SF.

**Table 3:** Cat eyes were intracamerally injected with LD and SF (*n* = 3).

Groups	XI		XII		XIII	
Eye	Right	Left	Right	Left	Right	Left
LD con. (%)	2	0	2	2	2	0
SF con. (mg/L)	0	0	0	100	0	100

Note: this table shows the concentrations of LD and SF. For example, in group XII, the left eye was intracamerally injected with 150 *μ*L of sterile solution containing 2% LD and 100 mg/L SF. 2% LD is the clinical concentration of intracameral anaesthesia.

## Data Availability

The datasets used and/or analyzed during the current study are available from the corresponding author on reasonable request.
